# Dataset on the current state of air pollution in Bussau-Guinea Bussau: A diagnostic approach

**DOI:** 10.1016/j.dib.2018.09.032

**Published:** 2018-09-17

**Authors:** M.E. Emetere, M.L. Akinyemi, T. Oladimeji

**Affiliations:** a Department of Physics, Covenant University, Canaan land, P.M.B 1023, Ota, Nigeria; b Department of Chemical Engineering, Covenant University, Canaan land, Nigeria; c Department of Mechanical Engineering and Science, University of Johannesburg, APK, South Africa

**Keywords:** Air pollution, Aerosol, Bussau, Threats, Sustainability

## Abstract

Recent UN report shows that over 100,000 people die from air pollution. The general anthropoenic pollution from Sahara desert, fossil-fuel engines and bush-burning needs to be reduced to avoid natural accidents, regional climate change etc. A fifteen years dataset was obtained from the Multi-angle Imaging Spectro-Radiometer (MISR). The dataset generated from the primary dataset would assist to understand the state of air pollution over Bussau. It also serves as a reference to guide the choice of ground measuring equipments in the area. The aerosol constant and tunning constant over Bussau is 0.6694 and 0.1354 respectively. The maximum percentage aerosol loading is given as 14.8%.

**Specifications table**

**Table:** 

Subject area	Air Pollution
More specific subject area	Aerosol loading and Retention
Type of data	Table and figure
How data was acquired	Multi-angle Imaging Spectro-Radiometer (MISR).
Data format	Raw and analyzed
Experimental factors	Aerosol Optical Depth
Experimental features	Measurement at 550 nm
Data source location	Bussau
Data accessibility	Multi-angle Imaging Spectro-Radiometer

**Value of the data**

• The data gives a good background for further study on aerosol loading.
• The data provides technician necessary insight towards configurating sun-photometer over Bussau.
• The data helps to quantify the extent of air pollution.
• The data provides modeller necessary insight on aerosol loading and retention challenges over Bussau.

## 1 Data

The unique distribution of aerosols over the West African region in the last decade is evident in its diverse effects on life forms, regional meteorology [1] and the ozone layer. The satellite imagery of aerosols loading over West Africa from 2000 to 2015 show the implication of the impact of anthropogenic air pollution on human health, agricultural produce, thermal comfort and climate perturbations. Massive aerosols deposition into the atmosphere can contribute to the anthropogenic radiative forcing of climate [2], [3]. Moreover, the residence time of emitted aerosols show how significant the climatic influences of aerosols are most important in the immediate vicinity of the source regions [4], [5]. The current danger in most parts of West Africa is the non-availability of ground station to monitor aerosols properties and air pollution. Most research in the West African region is based on satellite observations.

The primary data was obtained from Multi-angle Imaging Spectro-Radiometer (MISR) i.e. found in Table 1. The tunning and atmospheric constants for fifteen was obtained using the West African regional scale dispersion model (WASDM) from the AOD dataset (Fig. 2, Fig. 3). The tunning and atmospheric constants are factors that determines the accuracy of ground instruments e.g. sun photometer [6], [7] and they are presented in Table 2. The secondary dataset i.e. aerosol loading was generated using the extended WASDM are presented in Table 3.

**Table 1:** Statistical analysis of AOD over research site.

	2000	2001	2002	2003	2004	2005	2006
Number of values	8	10	10	10	10	9	11
Minimum	0.26	0.25	0.29	0.1	0.23	0.23	0.28
Maximum	1.89	0.92	1.16	1.21	1.35	1.1	0.81
Mean	0.67	0.59	0.69	0.6	0.61	0.68	0.52
Standard error	0.18	0.08	0.11	0.11	0.11	0.09	0.06
Standard deviation	0.52	0.25	0.36	0.36	0.35	0.28	0.2
Coefficient of variation	0.77	0.43	0.52	0.6	0.57	0.41	0.38

	2007	2008	2009	2010	2011	2012	2013

Number of values	10	8	11	10	11	10	10
Minimum	0.29	0.47	0.25	0.21	0.25	0.3	0.28
Maximum	1.38	1.26	1.17	0.82	0.93	1.44	1.01
Mean	0.64	0.85	0.64	0.5	0.58	0.59	0.58
Standard error	0.13	0.1	0.09	0.07	0.06	0.11	0.07
Standard deviation	0.4	0.28	0.28	0.21	0.2	0.36	0.21
Coefficient of variation	0.62	0.33	0.45	0.41	0.34	0.6	0.36

**Fig. 1:**
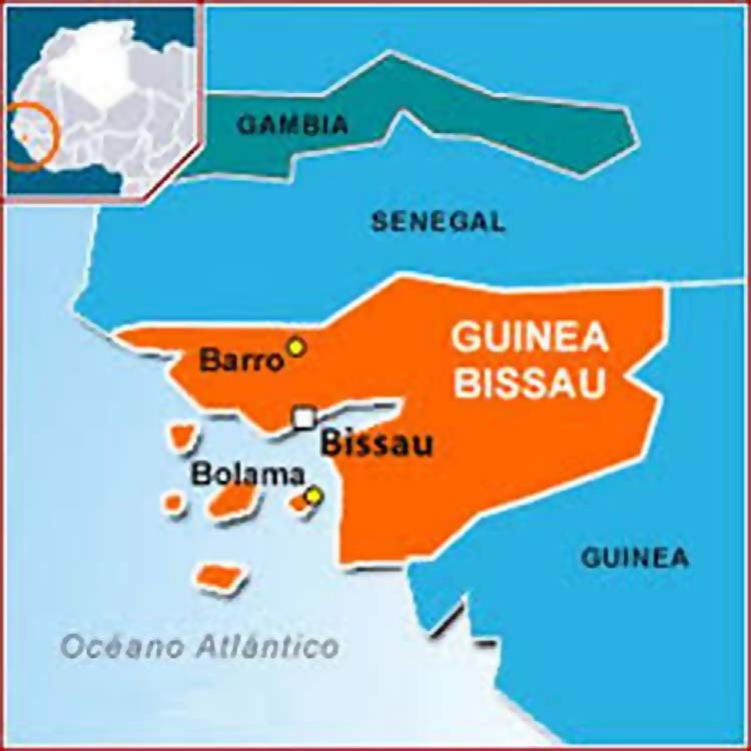
Geographical map of Bussau.

**Fig. 2:**
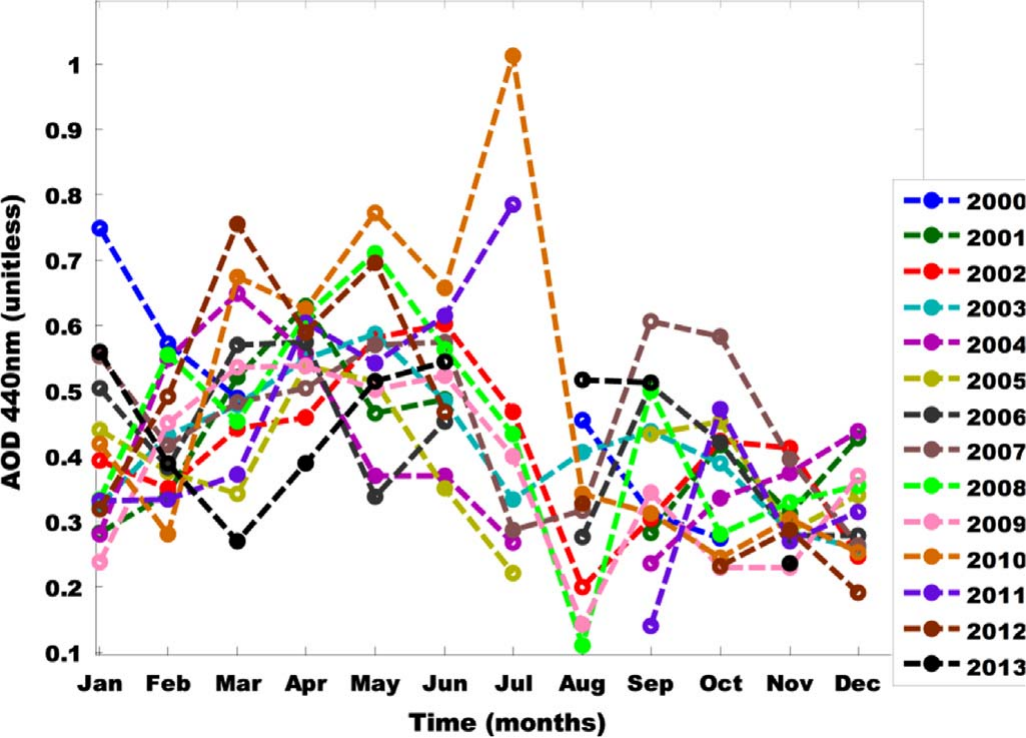
AOD pattern for Bussau 2000–2013.

**Fig. 3:**
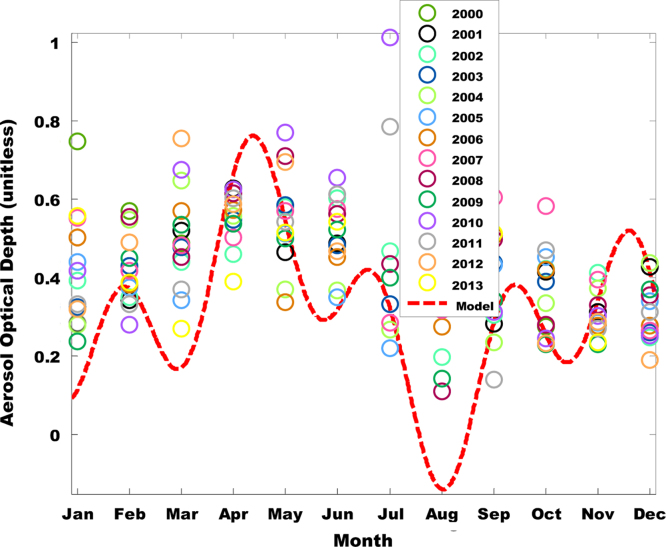
AOD for new model and MISR (Bussau, 2000–2013).

**Table 2:** Atmospheric constants over Bussua.

Location	$a_{1}$	$a_{2}$	$n_{1}$	$n_{2}$	α	Β
Bussau	0.6135	0.6694	0.1354	0.347	$\frac{\pi }{4}$	$\frac{\pi }{4}$

**Table 3:** Aerosol loading over Bussua.

**Month**	**2000**	**2001**	**2002**	**2003**	**2004**
**Jan**	0,734765985	0,867898608	0,841672788	0,856936364	0,869979572
**Feb**	0,80061927	0,86106121	0,850215591	0,833213223	0,798162243
**Mar**	0,827337404	0,809448697	0,82978118	0,814640664	0,758743056
**Apr**	0,89151364	0,774691358	0,829408069	0,800100507	0,807725806
**May**	0,89151364	0,840826496	0,796684202	0,787968924	0,856541712
**Jun**	0,89151364	0,823492533	0,824463389	0,811697549	0,864515229
**Jul**	0,89151364	0,89151364	0,827337404	0,867173139	0,877992029
**Aug**	0,831692315	0,89151364	0,883890793	0,831997952	0,89151364
**Sep**	0,866823132	0,869126523	0,871091816	0,835806237	0,881344544
**Oct**	0,874451401	0,850792057	0,830873735	0,843068365	0,865140445
**Nov**	0,89151364	0,865036736	0,846072742	0,86848423	0,848068955
**Dec**	0,89151364	0,852435706	0,878141098	0,874086438	0,836935581

**Month**	**2005**	**2006**	**2007**	**2008**	**2009**

**Jan**	0,834177041	0,82836097	0,790230852	0,858029946	0,876456644
**Feb**	0,844627417	0,858662629	0,837228415	0,795550012	0,827574285
**Mar**	0,855945708	0,787314931	0,818206985	0,824463389	0,808941199
**Apr**	0,803922839	0,796054829	0,825363734	0,768009384	0,818877544
**May**	0,816405132	0,866126012	0,799803484	0,729674845	0,823654787
**Jun**	0,864094476	0,828282503	0,815325887	0,780408196	0,820099605
**Jul**	0,875087824	0,89151364	0,862813336	0,832150497	0,846475382
**Aug**	0,89151364	0,871907092	0,865963016	0,888964075	0,886629993
**Sep**	0,839973512	0,833816485	0,797562242	0,814812231	0,860838673
**Oct**	0,833011487	0,845938152	0,807993282	0,873660625	0,878141098
**Nov**	0,87177234	0,872441588	0,855103199	0,860950039	0,877501938
**Dec**	0,866973432	0,870779603	0,870631879	0,853245413	0,857874754

**Month**	**2010**	**2011**	**2012**	**2013**	

**Jan**	0,839802114	0,862867282	0,861680155	0,817982841	
**Feb**	0,870755018	0,856841843	0,807279162	0,848305643	
**Mar**	0,748394685	0,848942503	0,705785374	0,871456164	
**Apr**	0,76688054	0,785540241	0,793773947	0,853678054	
**May**	0,728384275	0,813088767	0,745509595	0,820210232	
**Jun**	0,759873159	0,76289584	0,837179661	0,80656232	
**Jul**	0,616767219	0,718531231	0,89151364	0,89151364	
**Aug**	0,862597063	0,89151364	0,861282966	0,819378621	
**Sep**	0,877387621	0,888342508	0,89151364	0,802387938	
**Oct**	0,875224606	0,830307971	0,876925784	0,89151364	
**Nov**	0,866772933	0,87208606	0,870353517	0,874339508	
**Dec**	0,873703433	0,863723734	0,881344544	0,89151364	

## 2 Experimental design, materials and methods

Guinea Bissau is located on latitude 11°N to 12°N and longitude 14°W to 15°W. It is bounded within an approximate total area of 36,125 km^2^. Guinea Bissau geographical structure includes low coastal plain, Guinean mangroves and forest. Its climate is hot, dry, dusty harmattan haze in the dry season, and warm and humid in the wet season. Its wet season is from June to early October, and the dry season is from December to April. Bussua is located on longitude and latitude of −15.6° and 11.87° (Fig. 1).

The West African regional scale dispersion model (WASDM) for calculating aerosol loading over a region:.

(1) $$\psi \left(\lambda \right)={a_{1}}^{2}\cos\left(\frac{n_{1}\pi \tau (\lambda )}{2}x\right)\cos\left(\frac{n_{1}\pi \tau (\lambda )}{2}y\right)+\ldots \ldots {a_{n}}^{2}\cos\left(\frac{n_{n}\pi \tau (\lambda )}{2}x\right)\cos\left(\frac{n_{n}\pi \tau (\lambda )}{2}y\right)$$

a is atmospheric constant gotten from the fifteen years aerosol optical depth (AOD) dataset from MISR, n is the tunning constant, τ(λ) is the AOD of the area and ψ(λ) is the aerosol loading. The analysis of Eq. (1) was done using the C++ codes.

The value of the atmospheric and tuning constant for fifteen years was determine using Eq. (1) over fifteen years data (Fig. 1, Fig. 2). The statistical analysis of the AOD over the research area is shown in Table 1. The value atmospheric and tuning constant i.e. obtained from the comprehensive dataset is shown in Table 2 and the curve fitting technique is shown in Fig. 1, Fig. 2. The secondary dataset i.e. aerosol loading was generated using the extended WASDM (shown in Eq. (1)) are presented in Table 3. The percentage of the highest aerosol loading is shown in Table 4. It is calculated by finding the percentage increase between two consecutive years.

**Table 4:** Percentage of increase of aerosols loading over Nouakchott.

Year	2001	2008	2009	2011
Percentage (%)	10.9	8.1	2.0	14.8
